# Salaries of Poor-Law Medical Officers

**Published:** 1914-05-02

**Authors:** 

**Affiliations:** Hon. Secretary Poor-Law Medical Officers' Association of England and Wales. 34 Copthall Avenue, London Wall, E.C.


### THE EDITOR'S LETTER-BOX.

### Salaries of Poor-Law Medical Officers.

*To the Editor of* The Hospital.

Sir,—I have read with interest your criticism of my letter published in your issue of the 11th ult., and am quite familiar with your line of reasoning. The arguments you adduce I have often heard repeated at professional gatherings when this subject has been considered, but they have never carried conviction to my mind, nor do I believe they have been accepted by any authoritative body representing the medical profession. The conditions in Bermondsey are not dissimilar to those in other parts of the Metropolis, and I do not admit the great defect of the system under which outdoor relief is carried on by a staff of part-time district medical officers. I have had over thirty years' experience in an area quite as crowded as Bermondsey, and never found any difficulty in keeping in touch with my indoor colleagues with regard to patients of mine entering the infirmary; and I have always been ready to inform the infirmary medical officers—indeed, have repeatedly gone out of my way to do so—of anything that I considered it necessary for them to know touching the treatment of the case. The defects arising from having all the Poor-Law medical relief centred in the Union infirmary are, in my opinion, far more weighty. I am not prepared to deny that such a system may be more economical, and if the saving of money were the only thing to be considered, your arguments might be more cogent. But I do not think the ideal of Poor-Law administration is to sweep all Poor-Law patients into the infirmary, even if the rates may gain a little thereby. Most poor persons, I believe, prefer to be attended by an independent doctor, who may have also attended them privately in better times, and who is in the habit of attending their friends and neighbours who are more fortunate than themselves. The poor are not in love with a system that provides them with an official doctor whose duties are limited to attending only those who come upon the rates, and who is merely the assistant of the medical superintendent of the infirmary. Neither can I understand your eulogy of the extra responsibility put upon that officer. From my knowledge of Poor-Law medical work, I should have thought he had quite enough to do in attending to the indoor work of a large infirmary, and that his responsibility for the outdoor work could never be more than nominal.

It is a mistake to suppose that a whole-time official who attends only Poor-Law cases can have anything like as good a view of the whole requirements of the parish as a medical practitioner who attends all classes there. The latter is in a far better position to assist the medical officer of health with regard to protecting the general health. The present district medical officers do possess great facilities for observing the sanitary shortcomings of the whole parish; but the assistant medical officers, who are to succeed them, will only be cognisant of what appertains to one particular class. I am glad you admit that the provisions for the old officers in Bermondsey is not a generous one. If they were willing to accept the new offices on the terms offered, it would prove at any rate that the present district medical officers of Bermondsey were hardly up to the standard of their colleagues in other districts. The compensation under the Superannuation Act is a pure farce, and it is almost unthinkable that a Board like that of Bermondsey would be likely to ask for permission to add ten years to the time of service of their deposed officers. I can further assure you that I do not miss the point of the Local Government Board's desire to have the proposed new assistant medical officers appointed for a term of years. I was alluding to the effect of this requirement of the upper Board. Under it the assistant (district) medical officer would have a tenure of five years—at least, that is my opinion—and would not be in a position to be discharged at any time by the Guardians as an ordinary assistant indoor medical officer. If I am in error, however, it will only make more manifest the undesirability of these appointments in the eyes of the profession; and I doubt very much whether many good candidates will apply for them, even if the Bermondsey Guardians are permitted by the Local Government Board to pay to the full the salaries they propose.—I am, yours truly,

Major Greenwood,
Hon. Secretary Poor-Law Medical Officers' Association of England and Wales.

34 Copthall Avenue, London Wall, E.C.

[The point in favour of the Bermondsey scheme which we made, and which Major Greenwood does not seem to realise, is that a centralised staff for both outdoor and indoor work makes it easy for the chief administrative medical officer to decide which cases ought to be admitted to the infirmaries and which can be treated just as well at home. It enables him to fill his beds to the greatest advantage, and his first-hand knowledge of the home conditions gives him the power to discharge many of the cases earlier than he could do without such knowledge. The result would not be to sweep all cases into the infirmary, but it would rather tend to reduce the number admitted and the length of their stay. In his remarks upon the relations of the district medical officers to the medical officer of health Dr. Greenwood does not differentiate between the duties of the district medical officer, as such, and his duties as a private practitioner.—Ed. The Hospital.]

